# Quality of care for pregnant women and newborns—the WHO vision

**DOI:** 10.1111/1471-0528.13451

**Published:** 2015-05-01

**Authors:** Ӧ Tunçalp, WM Were, C MacLennan, OT Oladapo, AM Gülmezoglu, R Bahl, B Daelmans, M Mathai, L Say, F Kristensen, M Temmerman, F Bustreo

**Affiliations:** ^1^ Department of Reproductive Health and Research including UNDP/UNFPA/UNICEF/WHO/World Bank Special Programme of ResearchDevelopment and Research Training in Human Reproduction (HRP)World Health OrganizationGenevaSwitzerland; ^2^ Department of Maternal, Newborn, Child and Adolescent HealthWorld Health OrganizationGenevaSwitzerland; ^3^ Family, Women and Children's Health ClusterWorld Health OrganizationGenevaSwitzerland

In 2015, as we review progress towards Millennium Development Goals (MDGs), despite significant progress in reduction of mortality, we still have unacceptably high numbers of maternal and newborn deaths globally. Efforts over the past decade to reduce adverse outcomes for pregnant women and newborns have been directed at increasing skilled birth attendance.^1^, ^2^ This has resulted in higher rates of births in health facilities in all regions.^3^ The proportion of deliveries reportedly attended by skilled health personnel in developing countries rose from 56% in 1990 to 68% in 2012.^4^ With increasing utilisation of health services, a higher proportion of avoidable maternal and perinatal mortality and morbidity have moved to health facilities. In this context, poor quality of care (QoC) in many facilities becomes a paramount roadblock in our quest to end preventable mortality and morbidity.

QoC during childbirth in health facilities reflects the available physical infrastructure, supplies, management, and human resources with the knowledge, skills and capacity to deal with pregnancy and childbirth—normal physiological, social and cultural processes, but prone to complications that may require prompt life‐saving interventions. Research shows that it is necessary to go beyond maximising coverage of essential interventions to accelerate reductions in maternal and perinatal mortality and severe morbidity.^5^ Moreover, there is a complex interplay of experiences of mistreatment and lack of support that impact women's childbirth experiences and outcomes.^6^

Moving beyond 2015, the World Health Organization (WHO) envisions a world where ‘every pregnant woman and newborn receives quality care throughout pregnancy, childbirth and the postnatal period.’ This vision is in alignment with two complementary global action agendas conceptualised by WHO and partners in 2013–2014—'Strategies toward Ending Preventable Maternal Mortality (EPMM)' and ‘Every Newborn Action Plan (ENAP)’.^7^, ^8^ It is articulated at a critical time when the global community is developing the new Global Strategy for Women's, Children's and Adolescents' Health (2016–2030) for the post‐2015 Sustainable Development Goal era.^9^

Although indirect causes of maternal death are increasing (27.5% of maternal deaths), globally, over 70% of maternal deaths occur as a result of complications of pregnancy and childbirth such as haemorrhage, hypertensive disorders, sepsis and abortion.^10^ Complications of preterm birth, birth asphyxia, intrapartum‐related neonatal death and neonatal infections together account for more than 85% of newborn mortality.^11^ Therefore, the time of childbirth and the period immediately after birth are particularly critical for maternal, fetal and neonatal survival and well‐being. Effective care to prevent and manage complications during this critical period is likely to have a significant impact on reducing maternal deaths, stillbirths and early neonatal deaths—a triple return on investment.^12^ Within this critical period, quality of care improvement efforts would target essential maternal and newborn care and additional care for management of complications that could achieve the highest impact on maternal, fetal and newborn survival and well‐being. Based on the current evidence on burden and impact, the following specific thematic areas have been identified as high priority for this vision:^10^, ^11^, ^12^

1. Essential childbirth care including labour monitoring and action and essential newborn care at birth and during the first week;
2. Management of pre‐eclampsia, eclampsia and its complications;
3. Management of postpartum haemorrhage;
4. Management of difficult labour by enabling safe and appropriate use of medical technologies during childbirth;
5. Newborn resuscitation;
6. Management of preterm labour, birth and appropriate care for preterm and small babies;
7. Management of maternal and newborn infections.

To end preventable maternal and newborn morbidity and mortality, every pregnant woman and newborn need skilled care at birth with evidence‐based practices delivered in a humane, supportive environment. Good quality of care requires appropriate use of effective clinical and non‐clinical interventions, strengthened health infrastructure and optimum skills and attitude of health providers, resulting in improved health outcomes and positive experience of women and providers. Moreover, quality of care is considered a key component of the right to health, and the route to equity and dignity for women and children.^13^

So, what is quality of care? To underpin this vision, we need a common understanding of what it means. This WHO vision defines quality of care as ‘the extent to which health care services provided to individuals and patient populations improve desired health outcomes. In order to achieve this, health care needs to be safe, effective, timely, efficient, equitable, and people‐centred.’^14^, ^15^ Operational definitions for the characteristics of quality of care are defined in Box 1.

Box 1 **Operational definitions for the characteristics of QoC definition^14^, ^15^**

- *Safe—*delivering health care which minimises risks and harm to service users, including avoiding preventable injuries and reducing medical errors
- *Effective—*providing services based on scientific knowledge and evidence‐based guidelines
- *Timely—*reducing delays in providing/receiving health care
- *Efficient—*delivering health care in a manner which maximises resource use and avoids wastage
- *Equitable—*delivering health care which does not vary in quality because of personal characteristics such as gender, race, ethnicity, geographical location or socioeconomic status
- *People‐centred—*providing care which takes into account the preferences and aspirations of individual service users and the cultures of their communities

Quality of care is a multi‐dimensional concept. Therefore, a framework with important domains of measurement and pathways to achieve the desired health outcomes is required to identify the action points to improve the quality of care. Since the Donabedian model of quality of care for health facilities was proposed in 1988, WHO and others have developed strategic thinking to operationalise key characteristics of QoC, using different elements from the provision of care as well as the experience of care, integral to maternal and newborn care provided in the facilities.^15^, ^16^, ^17^, ^18^, ^19^ WHO has also advanced health systems thinking by identifying six building blocks—service delivery; health workforce; information, medical products, vaccines and technologies; financing, and leadership/governance—creating a structure from where health systems analysis and intervention points can be established.^20^

Building on these developments, the framework (Figure 1) conceptualises QoC for maternal and newborn health by identifying domains of QoC which should be targeted to assess, improve and monitor care within the context of the health system as the foundation. Health systems create the structure which enables access to quality care and allows for the process of care to occur along two important and inter‐linked dimensions of provision and experience of care.

**Figure 1:**
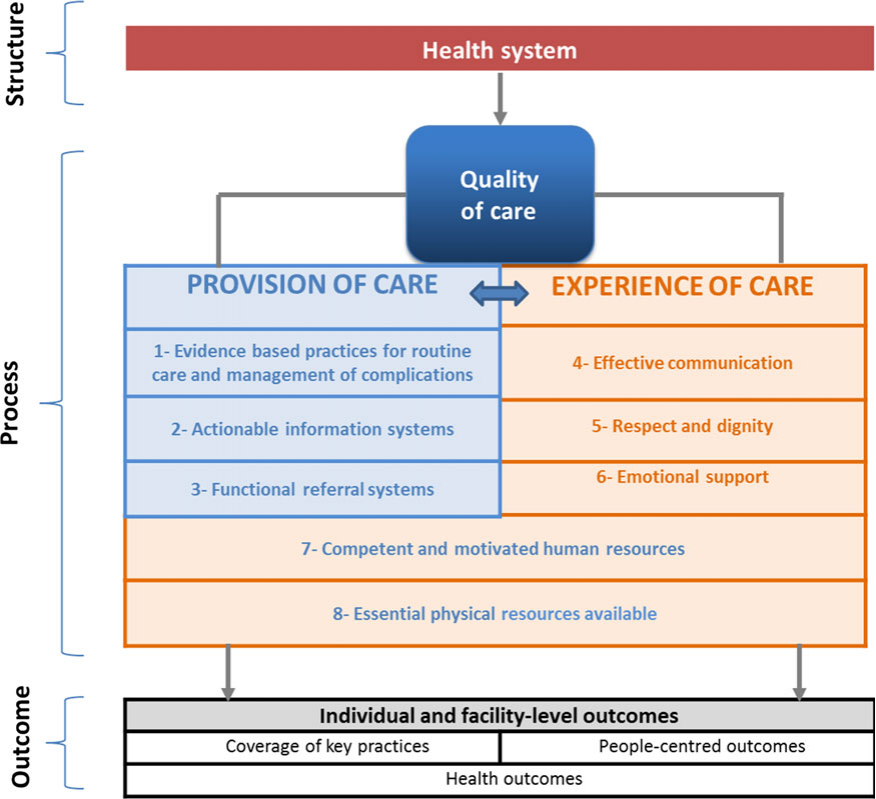
WHO Quality of Care Framework for maternal and newborn health.

Based on this framework, QoC for pregnant women and newborns in facilities requires competent and motivated human resources and the availability of essential physical resources. Also, evidence‐based practices for routine and emergency care, actionable information systems where record keeping enables review and audit mechanisms, and functional referral systems between levels of care should be in place. Experience of care includes firstly effective communication—a woman (or her family if required) should feel that she understands what is happening, what to expect and knows her rights. Secondly, she should receive care with respect and dignity. Thirdly, she should have access to the social and emotional support of her choice.

Improved QoC increases the likelihood of desired individual and facility‐level outcomes—health outcomes, coverage of key practices and people‐centred outcomes—with a focus on the identified high priority thematic areas described above. Although our framework focuses on the care provided in the facilities, it should be noted that communities and service users have a critical role in identifying their own needs and preferences, and in managing their own health. Perspectives of women, their families and communities, on the quality of maternity care services influence decisions to seek care and are essential components for creating a demand for and access to quality maternal and newborn services.^6^ Community engagement, therefore, is an important aspect to be considered.

A number of strategies that guide implementation efforts to improve QoC have been proposed. Many of these primarily focus on adapting interventions and working to overcome barriers to adaptation and implementation.^21^ However, these strategies do not always address the fundamental issue of achieving a balance between conformity to the evidence‐based practices and accommodating contextual differences, which underlies successful implementation. Moreover, without the appropriate tools and materials available in a user‐friendly format, health systems are less likely to implement an evidence‐based intervention, and, even if implemented, they may be suboptimal.

In this vision, WHO will use a QoC improvement strategy, an adaptation of the ‘Plan‐Do‐Study Act’ (PDSA) cycle model^22^ based on evidence synthesis, best practice and experience. This strategy provides a roadmap for continuous quality improvement. It starts by setting aims and building teams to achieve desired outcomes through implementation of evidence‐based change packages (individual, multi‐faceted and/or complex interventions depending on the context and the needs). It also incorporates capacity strengthening and other strategies to maximise the chances for sustaining the implementation.^23^, ^24^ In this context, quality improvement should achieve the standards set for both provision and experience of care.

Consolidating the framework and the improvement strategy described above, WHO will develop a comprehensive approach to provide guidance to global and national stakeholders to realise this vision. Figure 2 depicts how the WHO approach consolidates the QoC framework and improvement strategy, and highlights the identified strategic areas.

**Figure 2:**
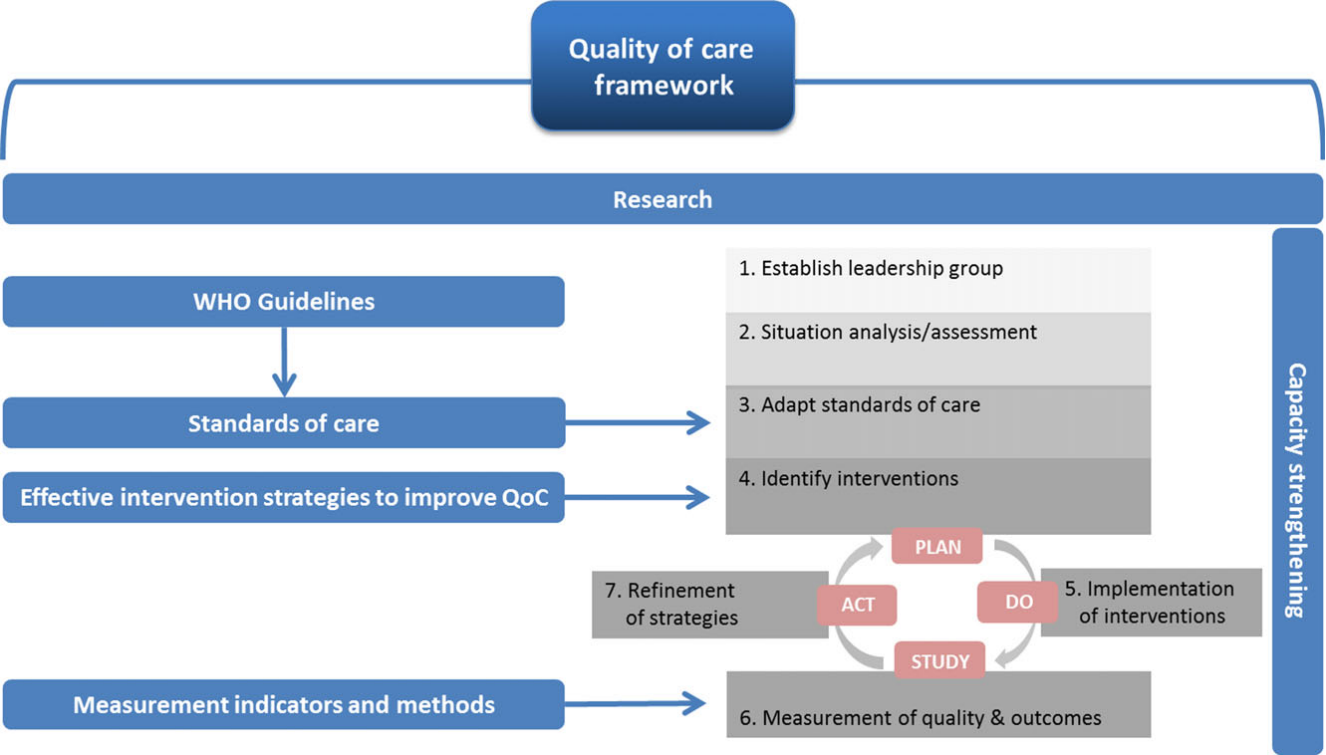
Developing the WHO approach – from framework to implementation.

In line with its organisational mandate (research, norms and standards, support for implementation, monitoring and evaluation),^25^ six strategic areas have been identified for WHO to contribute to ending preventable mortality and morbidity among mothers and newborns. The QoC definition and framework will inform this evidence‐based and systematic approach to (1) research, (2) guideline development, (3) standards of care, (4) identification of effective intervention strategies for quality improvement, (5) development of monitoring indicators at global, national and facility levels, and (6) capacity strengthening for quality improvement research, measurement and programming. Work in these strategic areas will support the maternal and newborn QoC improvement strategy and ensure implementation based on robust data, while including targeted country‐level capacity strengthening and technical support.

Given the progress made in MDG‐4 and MDG‐5 in the past 15 years, with increases in coverage of skilled attendance and essential intervention, the next phase should, in addition, target multiple domains of quality of care to reduce further the burden of preventable mortality and morbidity, integrated as part of the Global Strategy for Women's, Children's and Adolescents' Health.

## Disclosure of interests

None declared. Completed disclosure of interests form available to view online as supporting information.

## Contribution of authorship

The idea of this commentary was conceived by ÖT, AMG, MT and RB. ÖT, AMG, RB, WW, CM, OO, BD, MM, LS, FK, MT and FB all contributed the content and development of the article. All authors reviewed and agreed to the final version of this manuscript. All of the co‐authors are staff members at the World Health Organization.

## Details of ethics approval

No ethics approval required.

## Funding

None.
